# Forsythiaside A ameliorates bleomycin‐induced pulmonary fibrosis by inhibiting oxidative stress and apoptosis

**DOI:** 10.1002/iid3.70006

**Published:** 2024-08-22

**Authors:** Fan Yang, Qinqin Zhang, Xi Wang, Yingbo Hu, Suiqing Chen

**Affiliations:** ^1^ Henan University of Chinese Medicine Zhengzhou China; ^2^ Henan Key Laboratory of Chinese Medicine Resources and Chemistry Zhengzhou China; ^3^ Collaborative Innovation Center of Research and Development on the Whole Industry Chain of Yu‐Yao Henan University of Chinese Medicine Zhengzhou Henan Province China; ^4^ Co‐construction Collaborative Innovation Center for Chinese Medicine and Respiratory Diseases by Henan and Education Ministry of P.R. Henan University of Chinese Medicine Zhengzhou China

**Keywords:** A549, apoptosis, bleomycin, Forsythiaside A, oxidative stress, pulmonary fibrosis

## Abstract

**Background:**

Pulmonary fibrosis (PF) is a common clinically critical disease characterized by high morbidity and high mortality. Forsythiaside A (FA) is a phenylethanol glycoside component in *Forsythia suspensa*, which has anti‐inflammatory, antioxidant, and antiviral activities. However, the effects of FA on bleomycin (BLM)‐induced PF are unclear.

**Purpose:**

The present study explored the role of FA in the amelioration of oxidative stress and apoptosis in BLM‐induced PF as well as the possible underlying mechanisms, in vivo and in vitro.

**Methods:**

Network pharmacology was used to collect the effects of FA on BLM‐induced PF. Subsequently, further observation of the effects of FA on mice with PF by pulmonary pathological changes, transmission electron microscopy, real‐time polymerase chain reaction, Western blot analysis, immunofluorescence, and immunohistochemistry. An in vitro model was constructed by inducing A549 with transforming growth factor beta‐1 (TGF‐β1) to observe the effect of FA on epithelial cell apoptosis.

**Results:**

Network pharmacology predicted signaling pathways such as IL‐17 signaling pathway and Relaxin signaling pathway. The results of in vivo studies showed that FA ameliorated BLM‐induced PF through inhibition of fibrosis, modulation of apoptosis, and oxidative stress. In addition, FA promoted TGF‐β1‐induced apoptosis in A549 cells.

**Conclusions:**

The results of our study suggested that FA could protect mice against BLM‐induced PF by regulating oxidative stress and apoptosis as well as the Epithelial mesenchymal transition pathway.

## Graphical abstract

Our study demonstrated that Forsythiaside A (FA) has strong binding activity to Caspase 1, Caspase 3, Caspase 8, Fibroblast Growth Factor Receptor 1, Mitogen Activated Protein Kinase 14, and phosphatidylinositol 4,5‐bisphosphate 3‐kinase catalytic subunit gamma targets, and its significant amelioration of bleomycin‐induced pulmonary fibrosis (PF) may be related to its effects on PF by modulating the levels of inflammatory response, oxidative stress, and apoptosis in vivo, as well as regulating the Epithelial mesenchymal transition and hypoxia‐inducible factor ‐1 alpha (HIF‐1α)/vascular endothelial growth factor (VEGF)/transforming growth factor beta‐1(TGF‐β1) pathways. These positive effects of FA, may indicate its potential clinical for the treatment of PF.

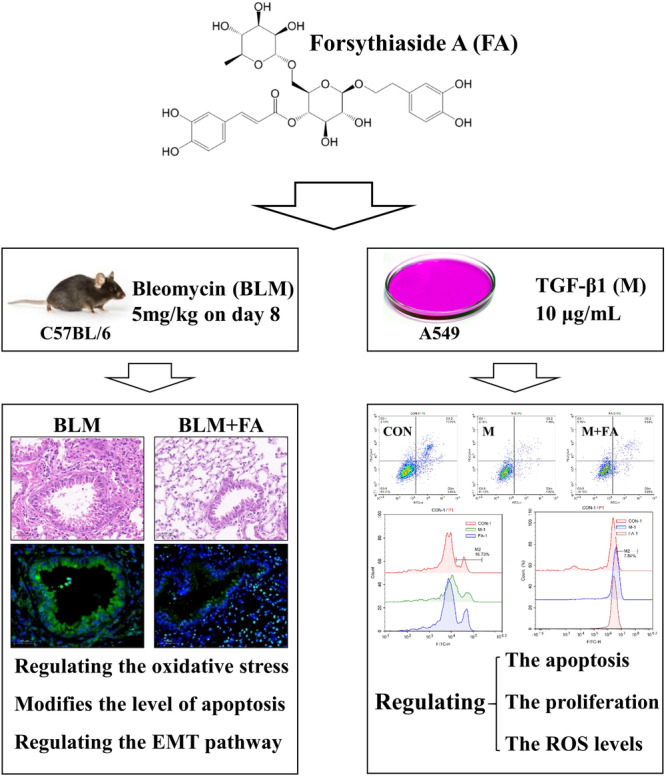

## 1 INTRODUCTION

Pulmonary fibrosis (PF) is a common clinically critical disease characterized by high morbidity and high mortality.^1^ PF poses a formidable clinical challenge, characterized by the fibrotic remodeling of lung tissue that significantly compromises respiratory function.^2^ Within the spectrum of contributors to PF, bleomycin (BLM)‐induced injury stands out as a pertinent model for unraveling the intricacies of fibrotic processes.^3^ BLM is an aminoglycopeptide antibiotic with good antitumor activity. However, the toxic side effects of BLM on the lungs limit its clinical application, the most serious of which is causing PF.^4^ BLM causes PF through mechanisms that affect collagen synthesis, oxidative stress, fibrotic cell proliferation, and apoptosis in lung tissue. The BLM‐induced mouse PF model is similar to human PF, characterized by interstitial fibrosis and collagen deposition in the mouse lung. So we chose BLM to construct a PF model.^5^

Currently, pyfenidone (PFD) and nintedanib are the only two drugs available for the treatment of idiopathic PF, but these two drugs often have serious side effects that can seriously affect the health of patients.^6^ Therefore, the study of PF drugs with good efficacy and low side effects is of great clinical significance. Forsythiaside A (FA) is a phenylethanol glycoside component in *Forsythia suspensa*, which has anti‐inflammatory, antioxidant, and antiviral activities. And studies have shown that FA can effectively inhibit lipopolysaccharide‐induced acute lung injury in mice.^7^ However, whether FA can attenuate BLM‐induced PF by modulating apoptosis and oxidative stress has not been investigated. Besides, A549 cells are type II‐like alveolar epithelial cells, which have become a commonly used in vitro model to study the mechanism of action associated with PF after induction using transforming growth factor beta‐1 (TGF‐β1).^8^ We explored this hypothesis in vivo and in vitro on BLM‐induced PF in mice and TGF‐β1‐induced A549 cells, and delved into the role and mechanism of FA against PF, and provided experimental support for further clinical treatment.

## 2 MATERIALS AND METHODS

### 2.1 Material and reagents

Pirfenidone (PFD) was purchased from Shanghai Yuanye Biotechnology Co., Ltd., and Bleomycin (BLM, CAS No. 9041‐93‐4) was purchased from Shanghai Macklin Biochemical Co., Ltd., FA (Chengdu Alfa Biotechnology Co., Ltd., CAS: 79916‐77‐1, purity ≥ 98%).

### 2.2 Data collection

The genes related to BLM‐induced PF were gained from GeneCards (https://www.genecards.org/) and OMIM (Online Mendelian Inheritance in Man, https://www.omim.org/); the genes related to FA were gained from Swiss Target Prediction (http://swisstargetprediction.ch/)and jvenn (http://jvenn.toulouse.inra.fr/app/example.html) was used to find FA and PF common target genes. Kyoto Encyclopedia of Genes and Genomes (KEGG) pathway analyses were analyzed by DAVID (Database for Annotation, Visualization and Integrated Discovery‌, https://david.ncifcrf.gov/tools.jsp). After analysis using the DAVID database, the top 15 pathways were selected based on the screening criteria of *p* Value less than 0.5 and the order of enrichment from small to large. KEGG pathways were performed by clusterProfiler, an R package used for the enrichment analysis of gene clusters. The component‐target‐pathway network of FA in BLM‐induced PF was built by Cytoscape 3.10.1.

### 2.3 Molecular docking

First, download the small molecule ligands of the compounds from PubChem (https://pubchem.ncbi.nlm.nih.gov/) and download the corresponding protein receptors from protein data bank (PDB, https://www.rcsb.org/). The Caspase 1 (CASP1) protein (PDB: 1NME), Caspase 3 (CASP3) protein (PDB: 1NME), Caspase 8 (CASP8) protein (PDB: 1QTN), Fibroblast Growth Factor Receptor 1 (FGFR1) protein (PDB: 1AWG), Mitogen Activated Protein Kinase 14 (MAPK14) protein (PDB: 1A9U), and phosphatidylinositol 4,5‐bisphosphate 3‐kinase catalytic subunit gamma (PIK3CG) protein (PDB: 1E7U) were used as the receptors. And protein receptors are imported into the SailVina final v1.0 platform. We will get the corresponding the pdbqt file, convert the pdbqt to a pdb format file through OpenBabel software, save the combined result as a pdb file, and then import it into PLIP (https://projects.biotec.tu-dresden.de/plip-web/plip/index). The binding site was determined by the website, and finally, the corresponding amino acid positions were marked by Discovery Studio Visualizer software.

### 2.4 Animals

All the experiments and procedures carried out as part of this study were approved by the Animal Ethics Committee of the Henan University of Chinese Medicine, Zhengzhou, China. A total of 40 male C57BL/6 mice (6−8 weeks, specific pathogen free, 18−22 g) were purchased from Zhejiang Vital River Laboratory Animal Technology Co., Ltd. (License: SCXK [ZHE] 20190001). The mice were housed in specific‐pathogen‐free‐grade animal laboratories at 24°C, alternating day and night, with food and water provided ad libitum. Adaptive feeding was used for the experiment after 1 week. The mice were randomly divided into five groups as follows: control (CON, *n* = 8), BLM (BLM, 5 mg/kg, *n* = 8), PFD (50 mg/kg, *n* = 8), low‐dose FA + BLM (FA‐L, 20 mg/kg, *n* = 8), and high‐dose FA + BLM (FA‐H, 40 mg/kg, *n* = 8). PFD, FA‐L, and FA‐H were intraperitoneally administered. The PF model was made using disposable intratracheal drops of 5 mg/kg BLM in the remaining 4 groups, except for the normal CON group, which was injected with an equal dose of saline. On the first day after modeling, each group was administered the corresponding drug by gavage, and equal volumes of distilled water were given to the normal and model groups, and the samples were taken and analyzed after 3 weeks of drug administration. Randomization was achieved using a computer‐generated random number table, and blinding procedures were employed to ensure unbiased group assignment and analysis. The timeline of the BLM‐induced PF model is shown in Figure 2A.

### 2.5 Histomorphological examination

Six mice were selected from each group. The Lung tissue was harvested under deep anesthesia and fixed in 4% paraformaldehyde for 24 h and embedded in paraffin, whereupon sections were made and stained with hematoxylin and eosin (H&E), Masson, and Sirius red. The images were viewed under a microscope (Nikon).

### 2.6 Lung coefficient

Eight mice were selected from each group. The lung tissue of the mice was collected, wiped clean with gauze, weighed, and lung coefficient was calculated. Lung coefficient (lung index) = lung mass/body mass × 100%.

### 2.7 Transmission electron microscopy

Three mice were selected from each group. The lung tissue was cut into 1 × 1 × 1 mm and fixed in 2.5% glutaraldehyde electron microscope fixative for 4 h, washed in PBS, and fixed in 1% osmium acid fixative for 1.5 h. The lung tissue was dehydrated step by step in graded ethanol, dehydrated in acetone, embedded in 812 epoxy resin overnight, and polymerized into ultrathin sections, stained in a saturated hydrogen peroxide acetate aqueous solution for 10 min, and stained in a lead citrate solution for 8 min, and then dried. The following ultrastructures were observed by an electron TEM1400 transmission electron microscope: erythrocyte (red blood cells [RBC]), endothelial cell (Enc), nucleus (N), mitochondrion (M), rough endoplasmic reticulum (RER), elastic lamina (EL), and smooth muscle cell (SMC).

### 2.8 ELISA analysis

Bronchoalveolar lavage fluid was collected and used to detect the levels of IL‐6 (E‐EL‐M0044, Elabscience Biotechnology Co., Ltd.). Take the size of soybean grains from the upper lobe of the left lung of mice, six in each group, weigh them and put them into EP tubes, add 9 times the weight of the tissue in saline, grind them with a high‐speed low‐temperature tissue grinder, and centrifuge the homogenized samples at 3000 r/min for 10 min. take the supernatant and then follow the instructions in the reagent kit. TGF‐β1 (E‐EL‐0162, Elabscience Biotechnology Co., Ltd.) and E‐Cadherin (E‐EL‐M0211, Elabscience Biotechnology Co., Ltd.) according to the respective manufacturer's instructions.

### 2.9 Enzymatic assay

The lung tissue of mice was rinsed with ice saline, six in each group, swabbed dry, and weighed into EP tubes. Nine times the weight of the tissue was added to the saline, and three grinding beads of 3 mm in diameter were added to each EP tube. The homogenized specimens were centrifuged at 3000 r/min for 12 min at 10 s/time for 6 consecutive times using a high‐speed cryogenic tissue grinder, and the supernatant was taken to measure the contents of malondialdehyde (MDA), total superoxide dismutase (T‐SOD), and glutathione peroxidase (GSH‐Px). The procedure was performed according to the instructions on the kit. The procedure was carried out according to the instructions on the kit.

### 2.10 Western blot analysis

Six mice were selected from each group, Proteins from the lung tissue were extracted with a mammalian protein extraction kit (Beijing Com Win Biotech Co., Ltd.) and quantified using a BCA protein assay kit (PC0020, Beijing Solarbio Science & Technology Co., Ltd.). A protein amount of 60 μg from each sample was loaded and separated using the SDS‐PAGE gel. Then, BSA was used to block the membrane for 1.5 h, followed by the addition of the primary antibodies for 2.5 h: B cell lymphoma/leukemia‐2 (Bcl‐2) (ab59348, Abcam), Bcl‐2‐associated X protein (Bax) (ab32503, Abcam), Heme Oxygenase‐1 (HO‐1) (A19062, ABclonal), Vimentin (ab92547, Abcam), NADPH Oxidase 4 (NOX4) (ab154244, Abcam), catalase‐1 (CAT‐1) (ab152687, Abcam), and β‐actin (Ac026, ABclonal). Then, the protein was washed four times with phosphate buffered saline with Tween‐20 (PBST) for 5 min each time. After that, secondary antibody (anti‐rabbit IgG HRP or goat anti‐mouse IgG HRP) was added and incubated for 1 h, washed four times with PBST for 5 min each time, and finally with PBS. The protein levels were quantified using the Bio‐Rad ChemiDocXRS+ system.

### 2.11 Quantitative real‐time **polymerase chain reaction** (qRT‐PCR) analysis

The left lung was selected, with three mice in each group. Total RNA was extracted using a total RNA extraction kit (R1200, Solarbio). Quantification was performed by NanoDrop™ One ultraviolet spectrophotometer (Thermo Scientific), followed by reverse transcription using the BeyoRTTM III First Strand (complementary DNA Synthesis Kit (D7178M, Beyotime) to obtain template DNA. Finally, the levels of Vimentin, HO‐1, Bcl‐2, Bax, Recombinant NOX4, and CAT‐1, were detected using QuantiNova™ SYBR green PCR kit (Qiagen). Table 1 shows the sequence of primers used for qRT‐PCR amplification.

**Table 1:** Sequences of the primers for qRT‐PCR.

Gene	Primer sequences (5′‐3′)
Mouse *Vimentin*	F: GCAGTATGAAAGCGTGGCTG R: CTCCAGGGACTCGTTAGTGC
Mouse *HO‐1*	F: GCTAAGACCGCCTTCCTGCT R: ACGAAGTGACGCCATCTGTGA
Mouse *Bcl‐2*	F: TGACTTCTCTCGTCGCTACCGT R: CCTGAAGAGTTCCTCCACCACC
Mouse *Bax*	F: GCCTTTTTGCTACAGGGTTTCAT R: TATTGCTGTCCAGTTCATCTCCA
Mouse *NOX4*	F: AGGAGCAACAAACCTGTCACCA R: CTGTATATCCATACTCTGCTGTGCC
Mouse *CAT‐1*	F: ACAAAGGTGTTGAACGAGGAGGA R: CTTAGGCTTCTCAGCGTTGTACTTG
Mouse *GAPDH*	F: CCTCGTCCCGTAGACAAAATG R: TGAGGTCAATGAAGGGGTCGT

Abbreviations: Bax, Bcl‐2‐associated X protein; Bcl‐2, B cell lymphoma/leukemia‐2; CAT‐1, catalase‐1; HO‐1, Heme Oxygenase‐1; NOX4, NADPH Oxidase 4; qRT‐PCR, quantitative real‐time polymerase chain reaction.

### 2.12 Immunohistochemistry

Immunohistochemical studies were performed to detect Vimentin, Collagen I, and TGF‐β1. Lung tissues from three mice were selected from each group; fresh lung tissue was taken and immersed in 4% paraformaldehyde fixative for 24 h. The tissues were then embedded in paraffin, cut into 5‐mm‐thick segments, deparaffinized, and dehydrated. Endogenous peroxidases were inactivated using 3% H_2_O_2_, followed by Goat Anti‐Rabbit IgG H&L (horseradish peroxidase). Hematoxylin was used as a counterstain, and neutral balsam was used to mount the sections for observation. Under the electron microscope, the tan area showed positive expression.

### 2.13 Flow cytometry (FCM) analysis of A549 cells

A549 cells in 6‐well plates at a density of 25,000 cells/well. A549 cells were cultured according to a previously published procedure, and in addition to a normal CON, the cells were treated with M (10 μg/mL TGF‐β1) and FA (10 μM FA + 10.0 μg/mL TGF‐β1) groups. The cellular levels of apoptosis and ROS were subsequently measured by FCM.

The apoptosis of A549 cells was assessed by Annexin V‐PE/7‐AAD according to the instructions (V‐IF488/PI, G1513, Servicebio). A549 cell proliferation was detected according to the instructions (G1601, Servicebio). The ROS level of A549 was assessed with 2′,7′‐dichlorodihydrofluorescein diacetate (DCFH‐DA, CA1410, Solarbio). A549 cells were stained with 1 μM DCFH‐DA and were then incubated for 20 min with cellular analysis by FCM.

### 2.14 Data analysis

The data were analyzed by IBM SPSS 26.0 software, and GraphPad Prism 8.0.1 software was used to draw the graphs. Results were expressed as the mean ± standard deviation. Statistical significance was assessed using one‐way analysis of variance and least significant difference tests with 95% confidence intervals for the comparison of each experiment with its respective CON group. *p* < .05 indicates a significant difference, and *p* < .01 indicates a highly significant difference in results.

## 3 RESULT

### 3.1 Effects of FA on BLM‐induced PF based on network pharmacology

**Figure 1:**
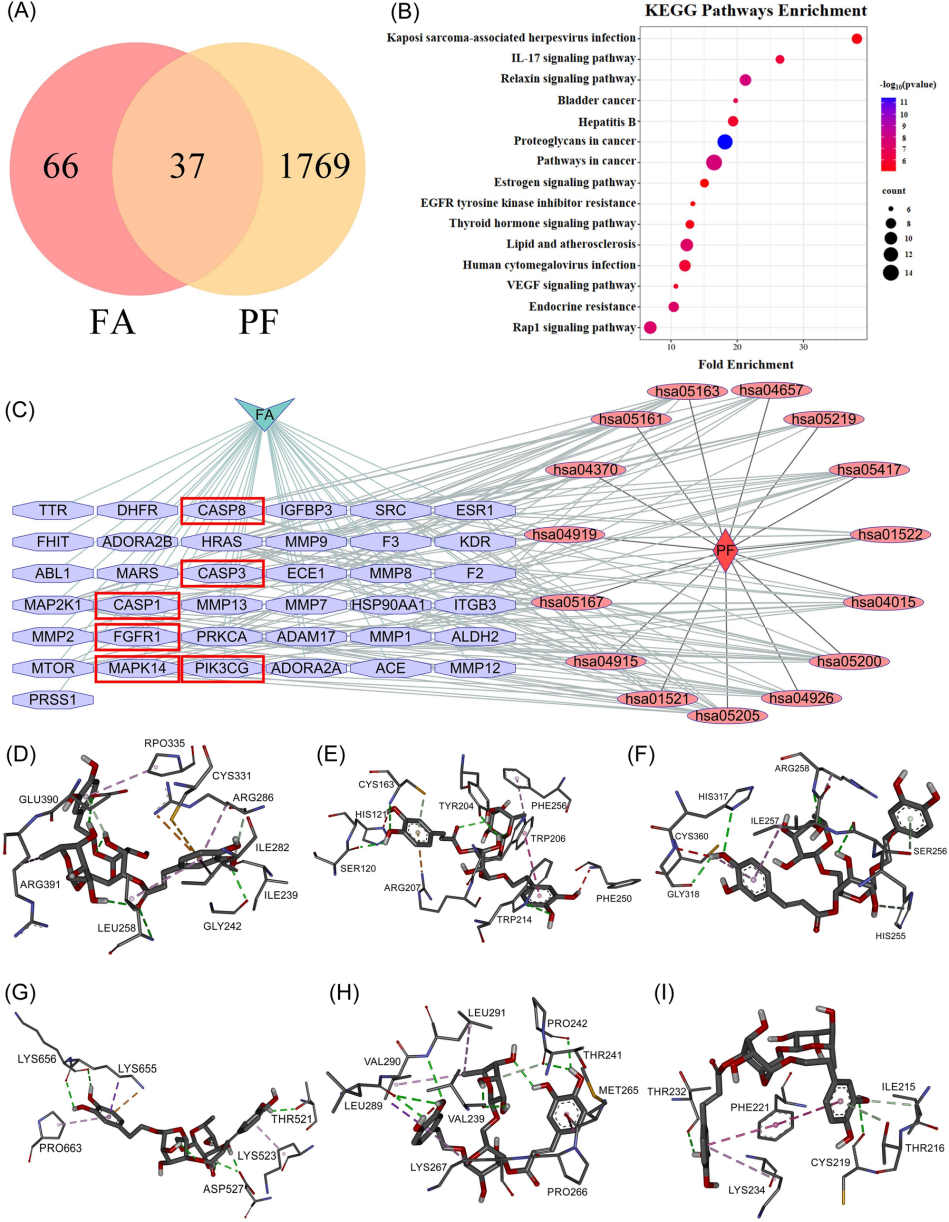
Effects of FA on BLM‐induced PF based on network pharmacology. (A) The target genes of FA in BLM‐induced PF were identified by jvenn. (B) KEGG enrichment analysis of FA in BLM‐induced PF. (C) The component‐target‐pathway network analysis of FA in BLM‐induced PF. Component FA in diamonds, targets intersecting disease and component in octagons, KEGG pathway designations in ellipses, disease PF in V's, and key protein targets selected in red boxes. (D−I) Strong binding forms of FA to six key targets of action identified by molecular docking. In order, these are FA with CASP1, CASP3, CASP8, FGFR1, MAPK14, and PIK3CG. BLM, bleomycin; CASP1, Caspase 1, CASP3, Caspase 3, CASP8, Caspase 8, FA, Forsythiaside A; FGFR1, Fibroblast Growth Factor Receptor 1, MAPK14, Mitogen Activated Protein Kinase 14; PIK3C, phosphatidylinositol 4,5‐bisphosphate 3‐kinase catalytic subunit gamma. KEGG, Kyoto Encyclopedia of Genes and Genomes.

A total of 1806 genes related to PF were collected from GeneCards, and a total of 103 target genes related to FA were obtained from Swiss Target Prediction. Further, 37 target genes of FA for the treatment of PF were found through jvenn (Figure 1A). The 37 target genes of FA for the treatment of PF were subjected to KEGG analysis. The results of the KEGG metabolic pathway enrichment analysis are shown in Figure 1B. By using Cytoscape 3.10.1, intersecting targets of FA and PF were analyzed to construct a component‐target‐pathway network for FA treatment of PF (Figure 1C). From the above component‐target‐pathway network analysis, we identified 6 key protein targets, including CASP1, CASP3, CASP8, FGFR1, Mitogen Activated Protein Kinase 14 (MAPK14), and phosphatidylinositol 4,5‐bisphosphate 3‐kinase catalytic subunit gamma, which could be the major biological hubs responsible for the anti‐pulmonary‐fibrosis function of FA. The binding energy value of less than 0 suggests that the ligand molecules can spontaneously bind to the receptor protein. The molecular docking results showed that the binding energies of FA to the targets were all lower than −5 kcal/mol, indicating that FA has good binding affinity to the six hub protein targets, CASP1, CASP3, CASP8, FGFR1, MAPK14, and PIK3CG, and the binding free energies were in the following order: −7.1, −7.5, −6.6, −6.9, −7.1, and −6.8 kcal/mol. The docking patterns of the FA and hub proteins are shown in Figure 1D−I. This docking study shows that FA could potentially bind to six hub targets, including CASP1, CASP3, CASP8, FGFR1, MAPK14, and PIK3CG, which may trigger synergistic effects among different signaling pathways. This suggests the use of FA in the treatment of PF and provides data support for further experimental design.

### 3.2 Effects of FA on histopathology and functions of lung in BLM‐induced PF mice

**Figure 2:**
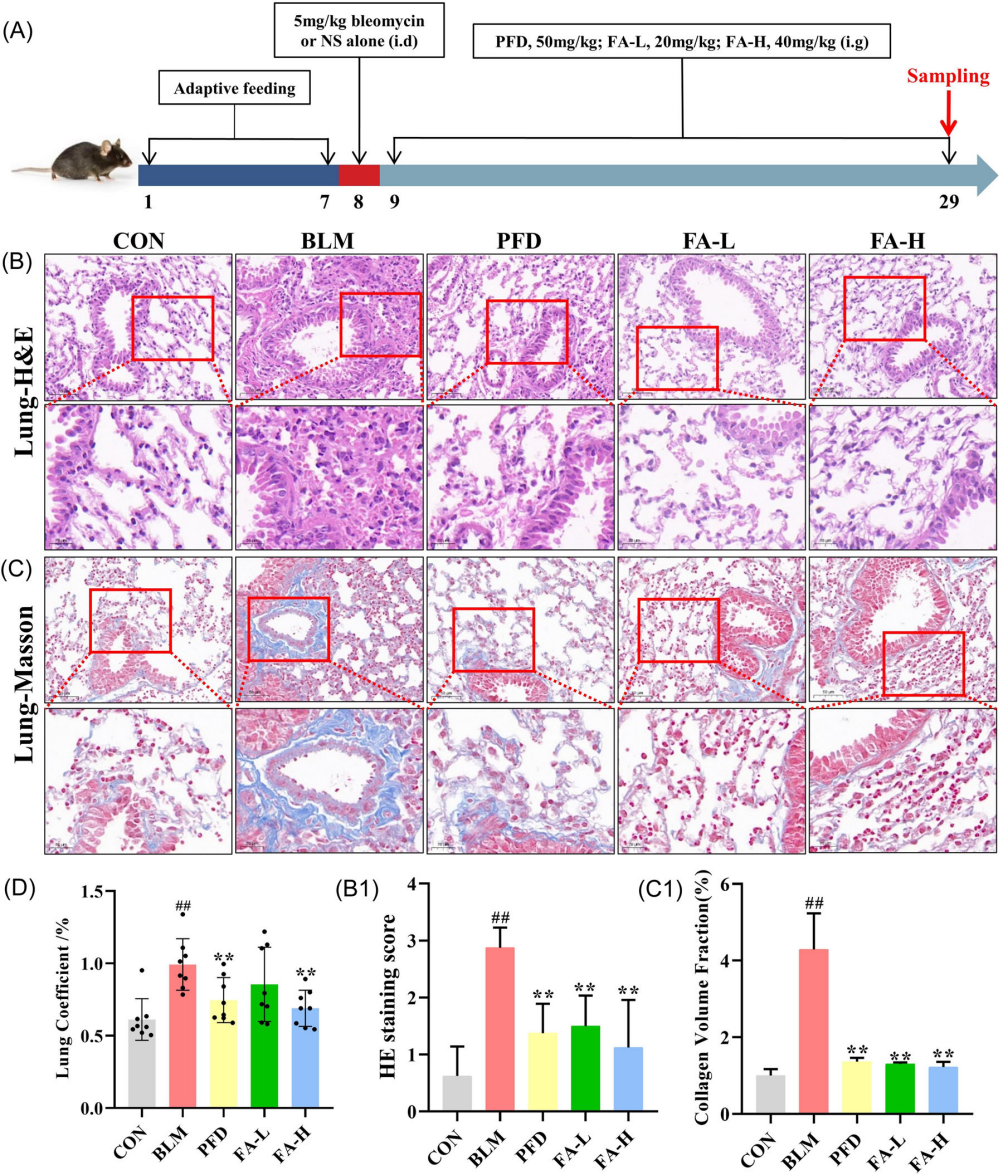
(A) Timeline of the BLM‐induced PF model male C57BL/6 mice were given 5 mg/kg BLM or saline via the disposable intratracheal drip on Day 8. PFD, FA‐L, FA‐H, or distilled water were administered from Day 9, and samples were collected after 3 consecutive weeks. (B) H&E staining of lung tissue (×20 magnification). (B1) The score of H&E staining (*n* = 6 mice per group). (C) Masson staining of lung tissue (×20 magnification). (C1) Collagen volume fraction in Masson staining of lung tissue (*n* = 6 mice per group). (D) Lung coefficient (*n* = 8 mice per group). These data are expressed as mean ± SD, ^#^ *p* < .05, ^##^ *p* < .01, compared with the CON group, *p < .05, **p < .01 compared with the BLM group. H&E, hematoxylin and eosin. BLM, bleomycin; i.d, intratracheal drip; i.g, intragastric administration; PFD, pirfenidone; FA‐L, low‐dose Forsythiaside A; FA‐H, high‐dose Forsythiaside A; NS, normal saline.

**Figure 3:**
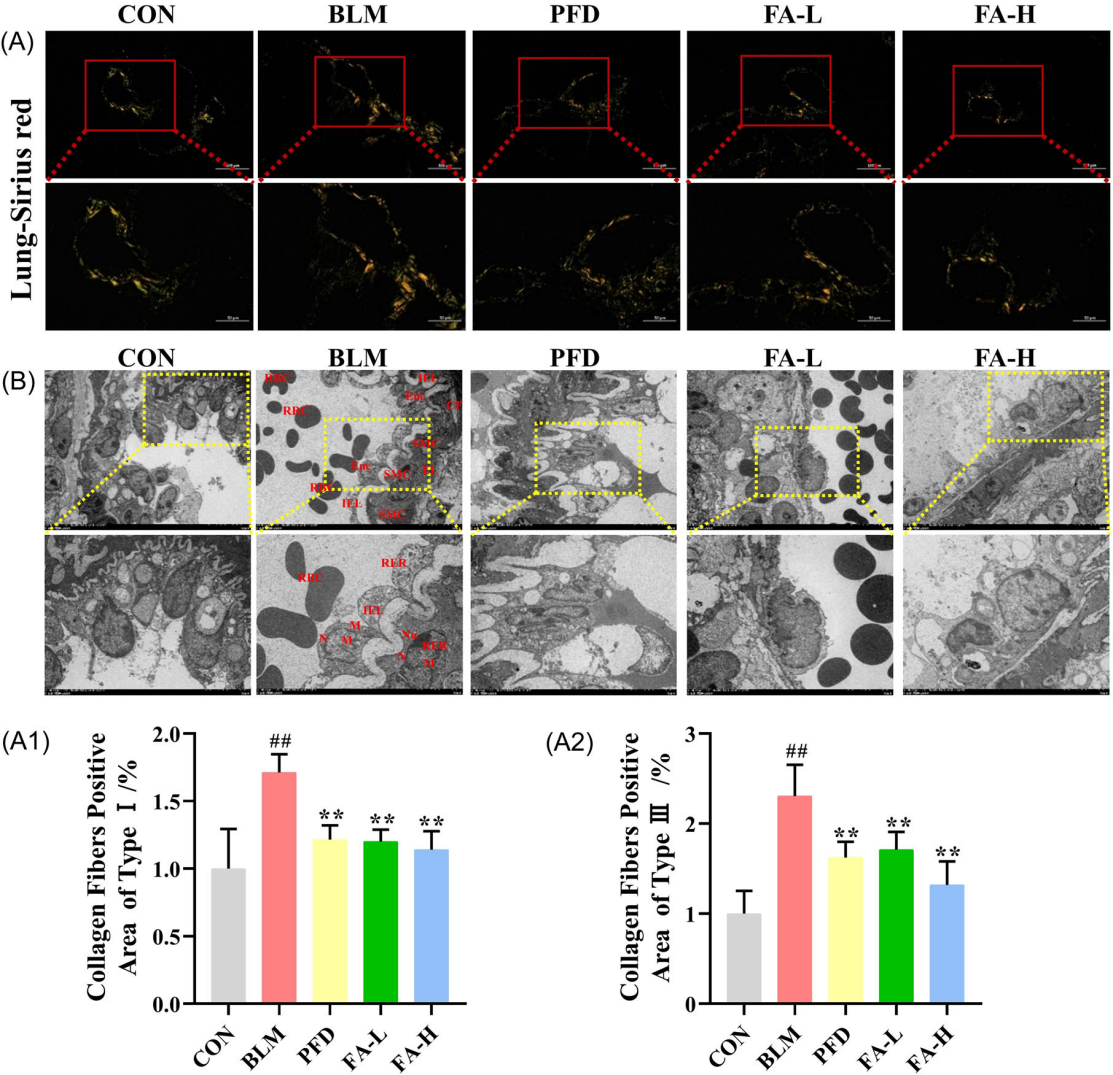
(A) Sirius red staining of lung tissue, x20 in the top row, x40 in the bottom row. (A1) The percent of area positive for type I collagen fibers (*n* = 6 mice per group). (A2) The percent of area positive for type III collagen fibers (*n* = 6 mice per group). (B) Lung tissue type II alveolar epithelial cell injury observed by transmission electron microscopy. These data are expressed as mean ± SD, ^#^ *p* < .05, ^##^ *p* < .01, compared with the CON group, *p < .05, **p < .01 compared with the BLM group. BLM, bleomycin; CON, control.

BLM administration was associated with significantly more inflammatory cell infiltration, collagen deposition, and type I and III collagen than the CON group. However, FA‐L and FA‐H attenuated lung tissues in mice with BLM‐induced PF (Figure 2B,C and 3A). The lung coefficient was significantly higher in the BLM group compared to the CON group, and FA could reduce the lung coefficient compared to the BLM group, with the FA‐H group significantly reducing the lung coefficient (Figure 2D).

### 3.3 Effects of FA on type II alveolar epithelial cell injury in BLM‐induced PF mice

As shown in Figure 3B, in the FA group compared to the M group, blood vessels and RBC were seen scattered in the lumen; Enc were moderately numerous, without obvious detachment, with obvious cytoplasmic edema, a small increase in heterochromatin in the N, and swelling and expansion of the mitochondria (M) and RER; the internal EL was structurally continuous, with a slightly thinner thickness in the local area and a significantly lower electron density; SMC were irregularly arranged, slightly swollen, with uniform cytoplasmic density, the nucleus (N) had a visible nucleolus (Nu) structure; the mitochondria (M) and RER were swollen and dilated; collagen fibers (CF) and elastic membranes (EL) were visible.

### 3.4 Effect of FA on BLM‐induced lung injury in PF mice

**Figure 4:**
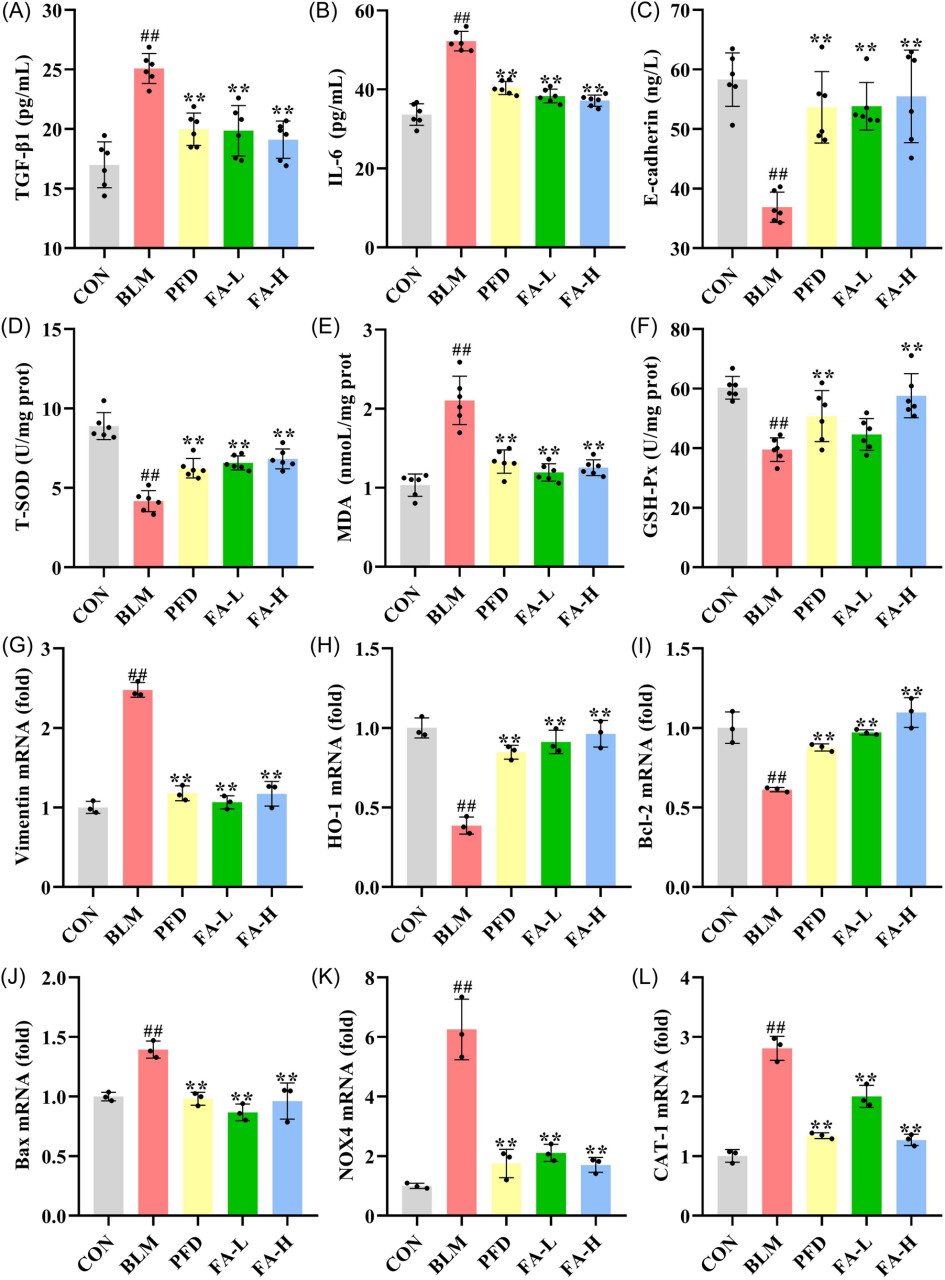
(A−C) Levels of TGF‐β1 and E‐cadherin in lung tissue and IL‐6 in BALF (*n* = 6 per group). (E−F) Levels of T‐SOD, MDA, and GSH‐Px in mice lung tissue (*n* = 6 per group). (G−L) Real‐time qPCR analysis of Vimentin, HO‐1, Bcl‐2, Bax, NOX4, and CAT‐1 (*n* = 3 per group). These data are expressed as mean ± SD, ^#^ *p* < .05, ^##^ *p* < .01, compared with the CON group, *p < .05, **p < .01 compared with the BLM group. BALF, bronchoalveolar lavage fluid; Bax, Bcl‐2‐associated X protein; Bcl‐2, B cell lymphoma/leukemia‐2; CAT‐1, catalase‐1; HO‐1, Heme Oxygenase‐1; NOX4, NADPH Oxidase 4. GSH‐Px, glutathione peroxidase; MDA, malondialdehyde; TGF‐β1, transforming growth factor beta‐1; T‐SOD, total superoxide dismutase.

As shown in Figure 4A−C, the results of the ELISA tests show that, compared with the CON group, the levels of TGF‐β1 and IL‐6 were significantly lower in the BLM group, and the levels of E‐cadherin were significantly higher in the BLM group, FA can reverse these levels. As shown in Figure 4D−F, T‐SOD and GSH‐Px levels were increased significantly and MDA levels were decreased significantly in the BLM group compared to the CON group, whereas T‐SOD and GSH‐Px levels were decreased significantly and MDA levels were increased notably in the FA‐L and FA‐H groups compared to the BLM group.

### 3.5 Effects of FA on apoptosis in BLM‐induced PF mice

**Figure 5:**
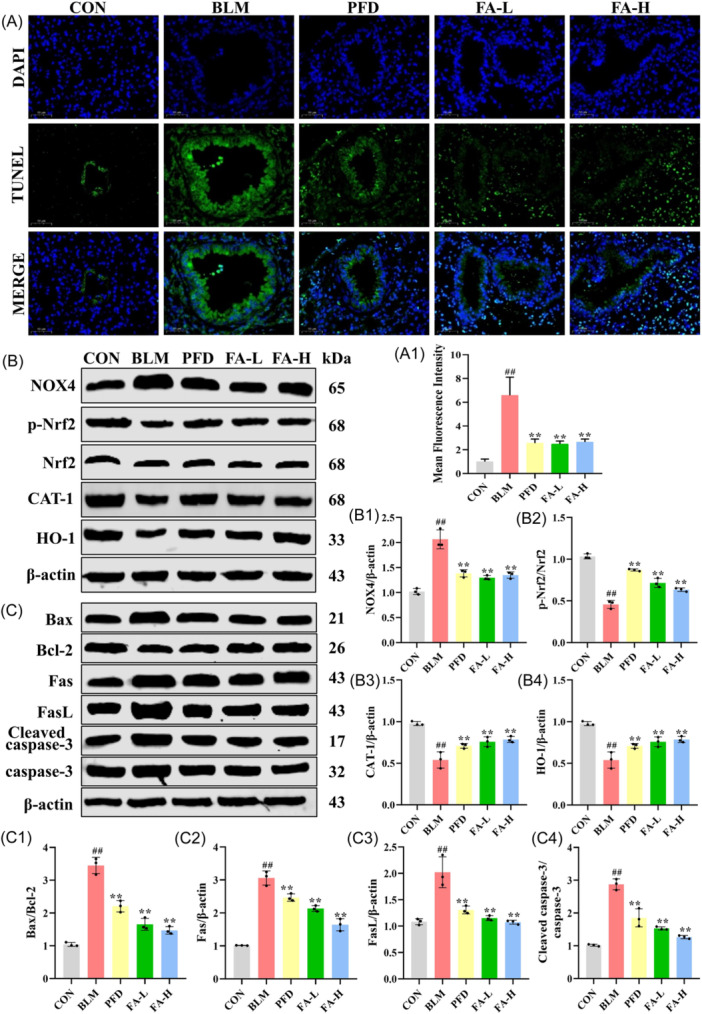
(A) Immunofluorescence staining of TUNEL in lung tissue (x200). (A1) Mean fluorescence intensity of TUNEL (*n* = 6 per group). (B) Western blot analysis of NOX4, p‐Nrf2, Nrf2, CAT‐1, HO‐1, and β‐actin protein in lung tissue. (B1‐B4) Relative expression of NOX4, HO‐1, CAT‐1, and p‐Nrf2/Nrf2 (*n* = 3 per group). (C) Western blot analysis of Bax, Bcl‐2, Fas, FasL, Cleaved caspase‐3, caspase‐3, and β‐actin protein in lung tissue. (C1‐C4) Relative expression of Bax/Bcl‐2, Fas, FasL, and Cleaved caspase‐3 (*n* = 3 per group). These data are expressed as mean ± SD, ^#^ *p* < .05, ^##^ *p* < .01, compared with the CON group, *p < .05, **p < .01 compared with the BLM group. Bax, Bcl‐2‐associated X protein; Bcl‐2, B cell lymphoma/leukemia‐2; CAT‐1, catalase‐1; HO‐1, Heme Oxygenase‐1; NOX4, NADPH Oxidase 4.

**Figure 6:**
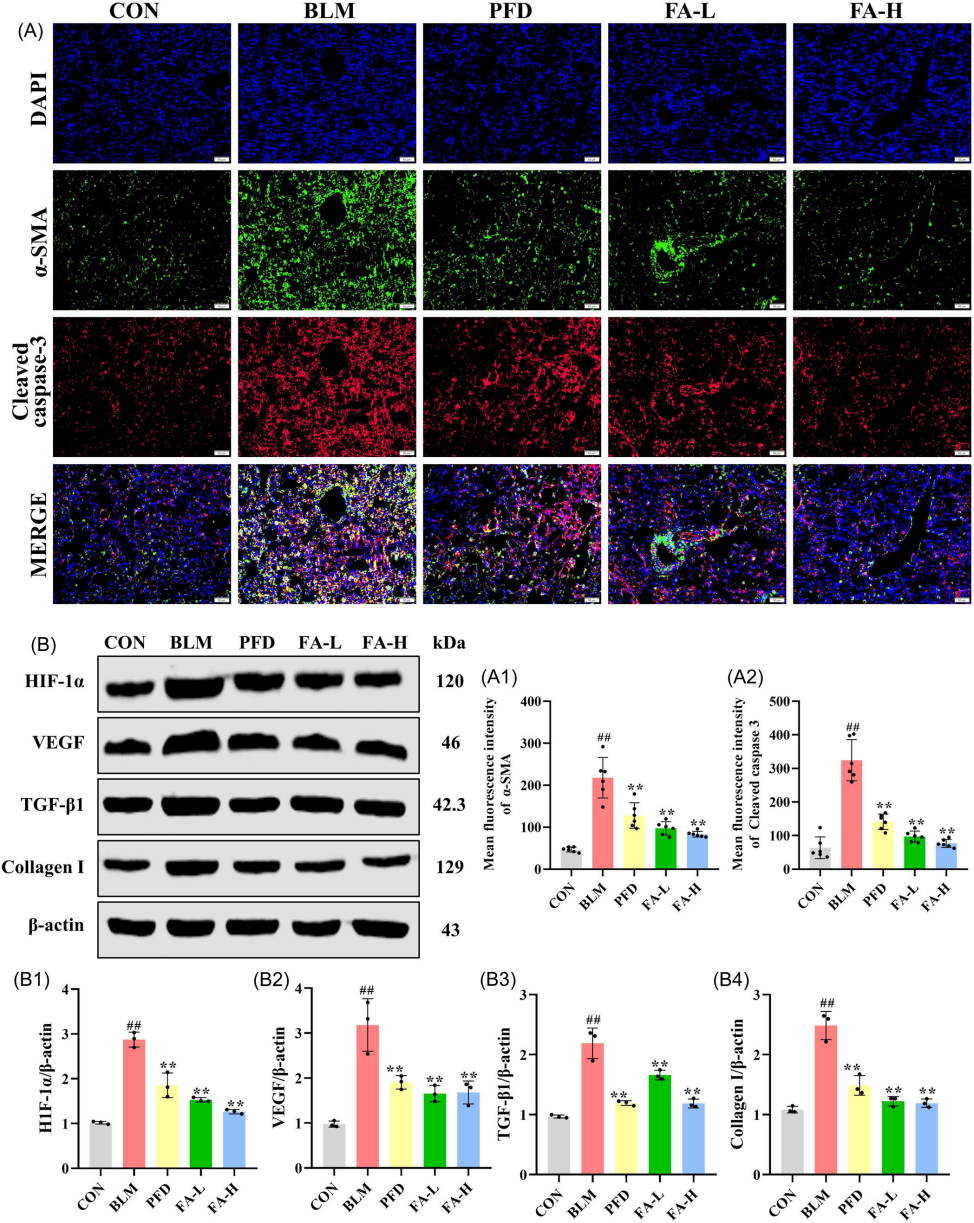
(A) Immunofluorescence staining of α‐SMA and Cleaved caspase‐3 in lung fibroblast (x200). (A1) Mean fluorescence intensity of α‐SMA (*n* = 6 per group). (A2) Mean fluorescence intensity of Cleaved caspase‐3. (B) Western blot analysis of HIF‐1α, VEGF, TGF‐β1, Collagen I, and β‐actin protein in lung tissue. (B1‐B4) Relative expression of HIF‐1α, VEGF, TGF‐β1, and Collagen I (*n* = 3 per group). These data are expressed as mean ± SD, ^#^ *p* < .05, ^##^ *p* < .01, compared with the CON group, *p < .05, **p < .01 compared with the BLM group. α‐SMA, α‐smooth muscle actin; HIF‐1α, hypoxia‐inducible factor ‐1 alpha;.

The mRNA levels of lung tissue were detected by qRT‐PCR (Figure 4G−L), the results of which showed that, compared with the CON group, the levels of Vimentin, Bax, NOX4, and CAT‐1 in the BLM group were notably boosted. FA resulted in the levels of these mRNAs being significantly lower in the coniferyl ester glycoside A group compared to the BLM group. The levels of HO‐1 and Bcl‐2 were significantly lower in the BLM group than in the CON group, whereas the levels of these mRNAs were significantly higher in the FA group than in the BLM group. As shown in Figure 5A, the levels of TUNEL were significantly increased in the lung tissues of mice with BLM‐induced PF compared with the CON group, and FA significantly reduced this level compared with the BLM group. The results of the western blot analysis show that in the BLM group, the levels of NOX4, Bax/bcl‐2, Fas, FasL, and Cleaved‐caspase 3/caspase 3 were substantially elevated in the lung tissues of the mice with PF as compared with the CON group, while the p‐Nrf2/Nrf2, CAT‐1, and HO‐1 levels were significantly reduced compared to the CON group, while FA‐L and FA‐H significantly reversed these levels compared to the BLM group (Figure 5B,C). Meanwhile, immunofluorescence results showed that Cleaved caspase‐3 levels were significantly elevated in the BLM group compared to the CON group, which was significantly reversed by FA (Figure 6A).

### 3.6 Effect of FA on BLM‐induced pro‐fibrotic factors in PF mice

**Figure 7:**
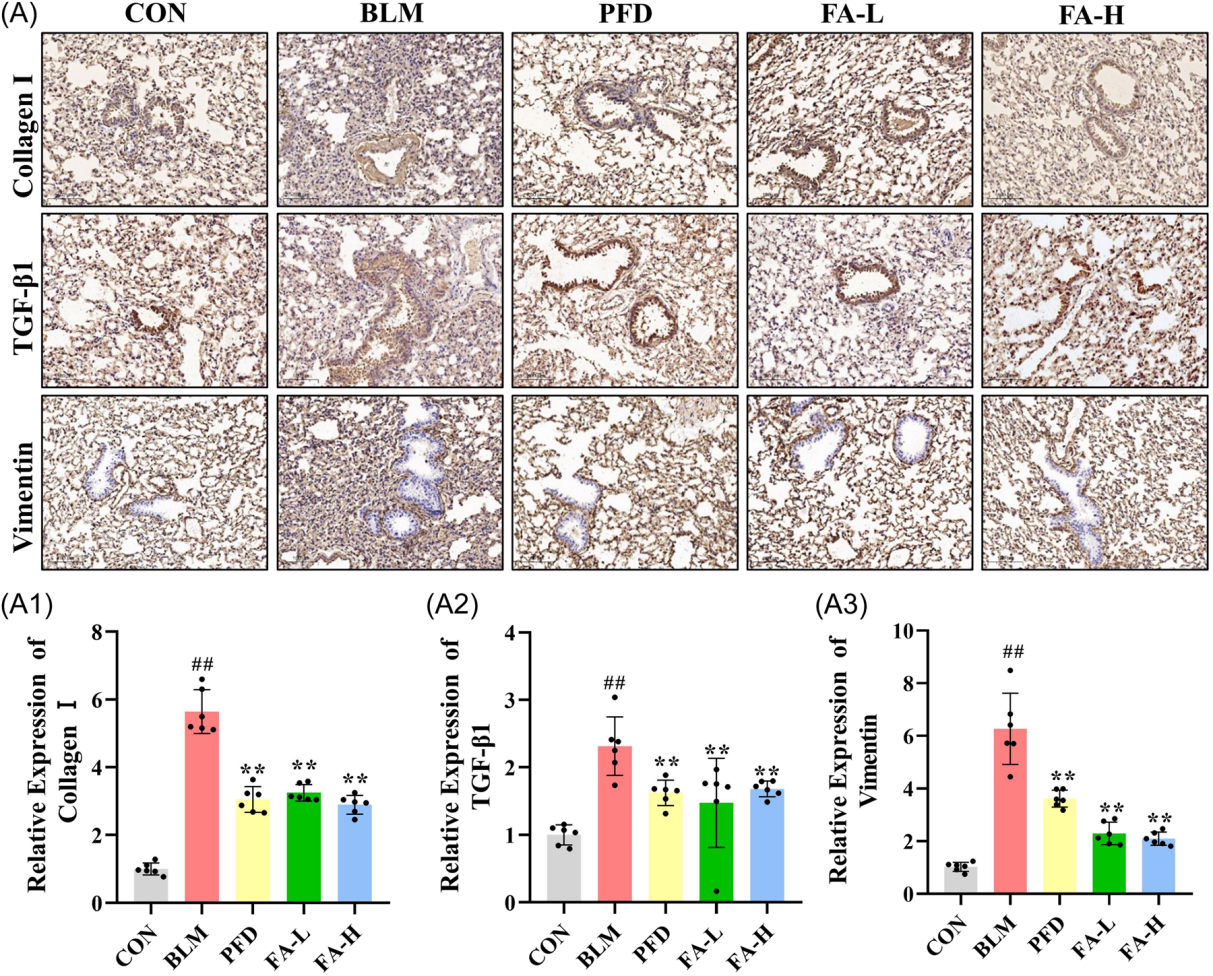
(A) Expression of Collagen I, TGF‐β1, and Vimentin in the lung tissue. (A1−A3) Relative expression of Collagen I, TGF‐β1, and Vimentin (*n* = 6 per group). These data are expressed as mean ± SD, ^#^ *p* < .05, ^##^ *p* < .01, compared with the CON group, *p < .05, **p < .01 compared with the BLM group. BLM, bleomycin; CON, control. TGF‐β1, transforming growth factor beta‐1.

Immunofluorescence results showed that α‐smooth muscle actin (α‐SMA) levels were significantly elevated in the BLM group compared with the CON group, whereas FA significantly reversed the level (Figure 6A). Immunohistochemistry results showed that Collagen I, TGF‐β1, and Vimentin levels were significantly higher in the BLM group compared to the CON group, and FA reversed these levels (Figure 7A). The results of the western blot analysis show that in the BLM group, the levels of NOX4, hypoxia‐inducible factor ‐1 alpha (HIF‐1α), vascular endothelial growth factor (VEGF), TGF‐β1, and Collagen I were substantially elevated in the lung tissues of the mice with PF as compared with the CON group, while FA significantly reversed these levels compared to the BLM group (Figure 6B).

### 3.7 Effect of FA on TGF‐β1‐induced proliferation, apoptosis and ROS levels in A549 cells

**Figure 8:**
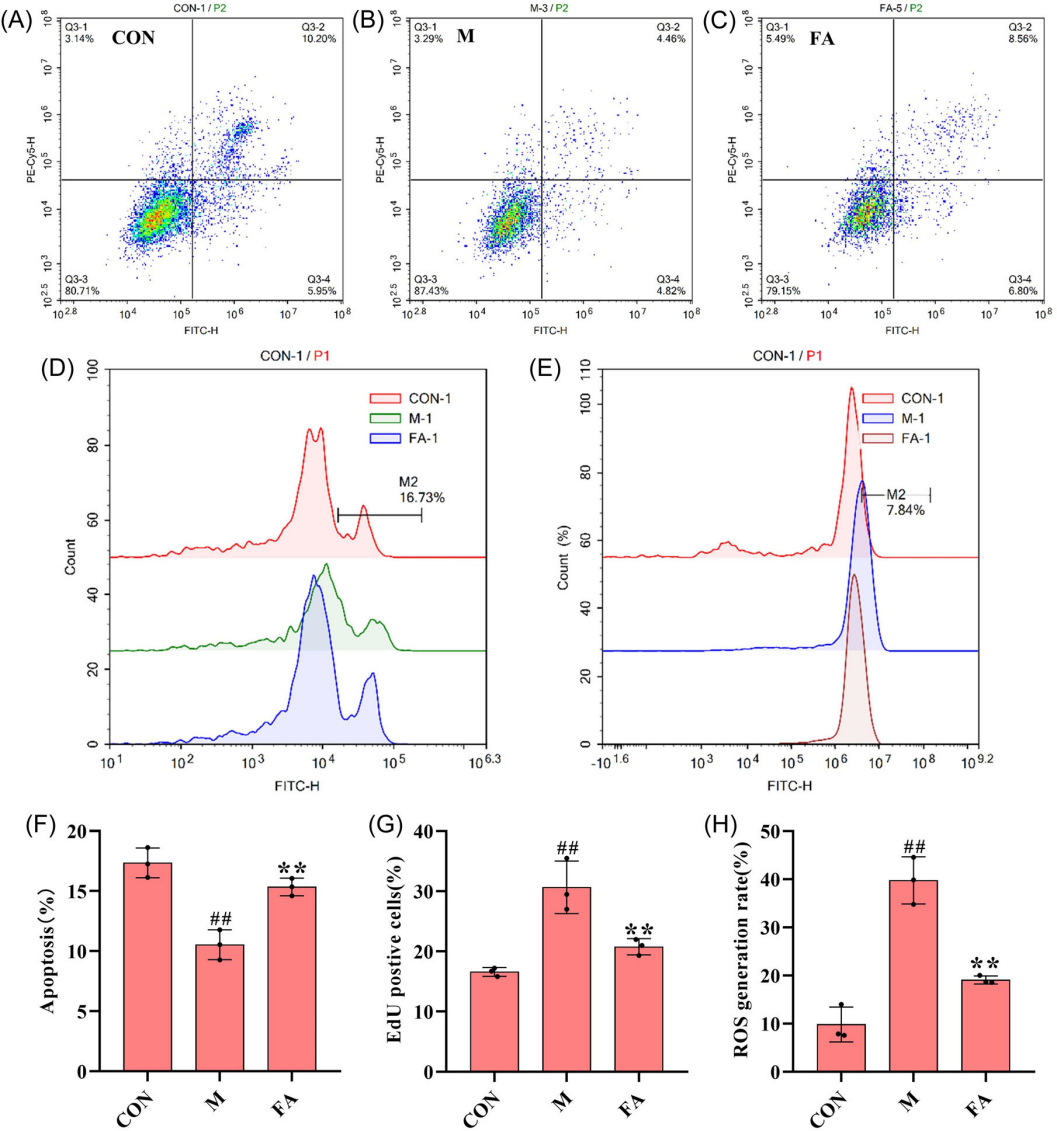
(A−C) Effect of FA on TGF‐β1‐induced A549 cells levels in apoptosis. (D) Effect of FA on TGF‐β1‐induced A549 cells levels in cell proliferation. (E) Effect of FA on TGF‐β1‐induced A549 cells levels in reactive oxygen species. (F−H) Relative levels of FA on TGF‐β1‐induced apoptosis, cell proliferation, and reactive oxygen species levels in A549 cells were detected by flow cytometry. These data are expressed as mean ± SD, ^#^ *p* < .05, ^##^ *p* < .01, compared with the CON group, *p < .05, **p < .01 compared with the BLM group. BLM, bleomycin; CON, control. TGF‐β1, transforming growth factor beta‐1.

**Figure 9:**
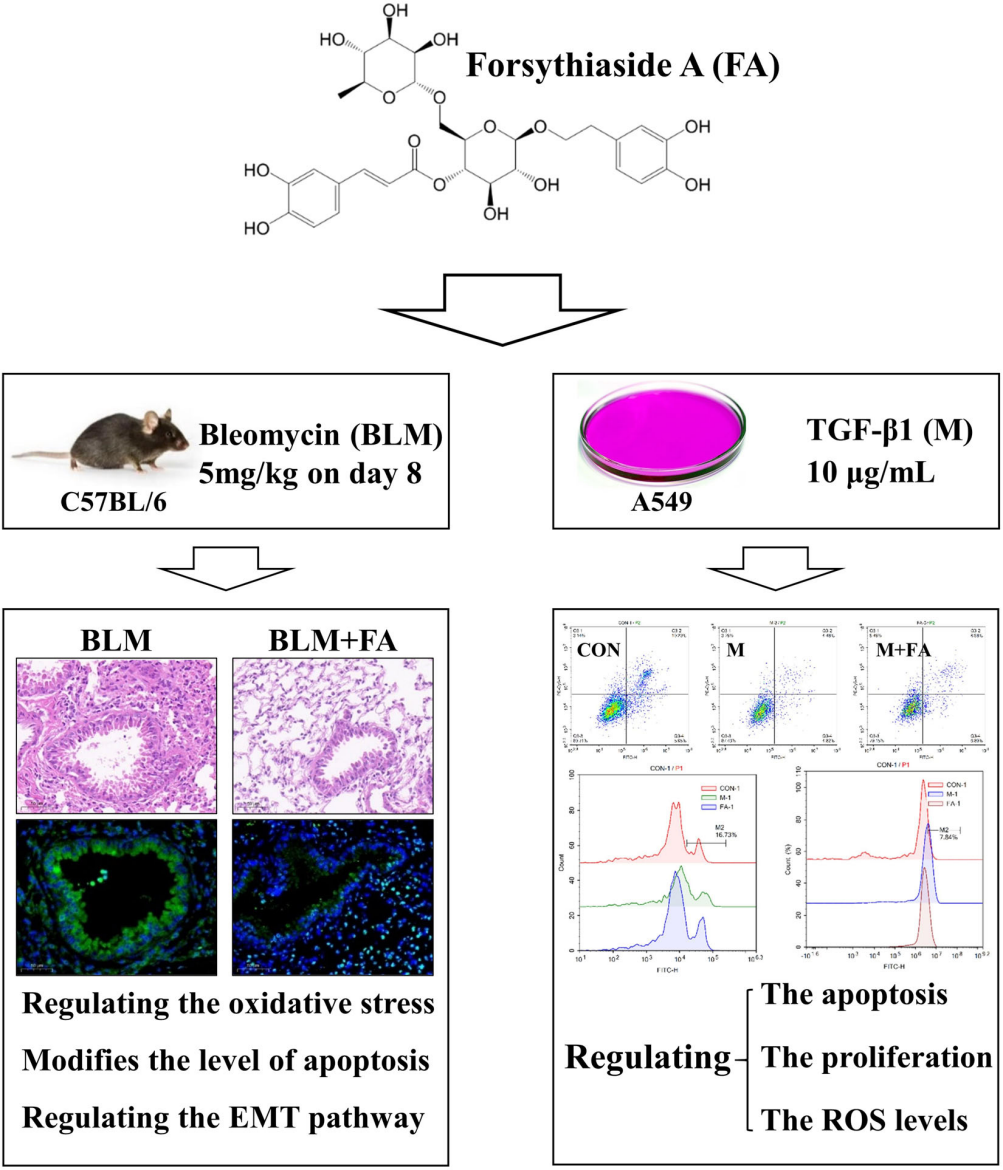
Mechanism of study on the intervention effect of Forsythiaside A (FA) on BLM‐induced PF in mice. BLM, bleomycin; CON, control; PF, Pulmonary fibrosis.

As shown in Figure 8 and 9, the levels of apoptosis were decreased and EdU positive cells and ROS were increased in TGF‐β1‐induced A549 cells, which could be reversed by FA.

## 4 DISCUSSION

PF is an immune‐mediated chronic inflammatory disease characterized by infiltration of inflammatory cells in the alveolar interstitium, proliferation of fibroblasts, and deposition of fibrous connective tissue in the alveolar interstitium, which lead to lung remodeling and scarring of lung.^9^, ^10^, ^11^ FA is a bioactive ingredient with pharmacological effects such as anti‐inflammatory and antioxidant.^12^ However, the interventional effect of FA on PF has not been reported. Therefore, in this study, we constructed an animal model of PF by disposable tracheal drip of BLM, and gave FA intervention to explore the interventional effect of FA on PF by observing the pathological injury of mice with PF, detecting the relevant indexes of oxidative stress, apoptosis, collagen deposition, and further exploring the mechanism of its action in interfering with PF.

In our study, we initially analyzed the potential targets of FA and PF with the use of network pharmacology. KEGG analysis showed that the potential mechanisms of action of FA in modulating PF were oxidative stress, apoptosis, and inflammation.^13^, ^14^ After constructing the component‐target‐pathway network, we identified six key targets of action: CASP1, CASP3, CASP8, FGFR1, MAPK14, and PIK3CG, of which CASP1, CASP3, and CASP8 influence the progression of PF by activating proinflammatory cytokines and initiating apoptotic cascades.^15^, ^16^ FGFR1 exacerbates PF by regulating the Epithelial mesenchymal transition (EMT) pathway,^17^ and MAPK14 plays a key role in oxidative stress and inflammatory responses.^18^ On the other hand, PIK3CG promotes fibroblast proliferation by regulating the PI3K/AKT signaling pathway.^19^ Meanwhile, molecular docking results showed that FA binds well to all six potential targets of action. Therefore, we hypothesized that FA might ameliorate PF by regulating oxidative stress, apoptosis, and EMT pathways.

Results of in vivo experiments showed that, compared with the CON group, the lung coefficients of mice in the BLM group were significantly higher, the alveolar walls were significantly thickened, a large number of inflammatory cell infiltration appeared in the mesenchyme, and CF deposition was significantly increased, which showed obvious destruction of alveolar structure and inflammatory infiltration, suggesting that a mice model of PF had been successfully induced and established by BLM.

Apoptosis is an important mechanism for the regulation of inflammation in the body and plays an important role in the pathogenesis of PF. Mitochondria, as important organelles in the apoptosis regulatory network,^20^ regulate some of the factors that are at the center of the process, such as cytochrome C, the Bcl‐2 family, and precursors of caspases, all of which are localized in the mitochondria. Bcl‐2 inhibits apoptosis by inhibiting the release of cytochrome C from mitochondria into the cytosol, while Bax induces apoptosis by binding to the permeability transition pore complex of the mitochondrial membrane and opening the pore, resulting in the release of cytochrome C.^21^, ^22^ Fas/FasL is an important signaling pathway in apoptosis, and the binding of Fas to the ligand FasL initiates lethal signaling through the Fas molecule, which ultimately induces a series of cellular characteristic changes, resulting in apoptotic cell death. During apoptosis, caspases can be cleaved by proteases to form activated caspases, and some caspases can sequentially activate other caspases to form a cascade of caspase reactions and promote apoptosis. Among the 14 known caspases, Caspase‐3, Caspase‐8, and Caspase‐9 have the closest relationship with apoptosis.^23^ Caspase‐9 is a key protease in the mitochondrial apoptosis pathway, and Caspase‐8 is a key protein in the death receptor pathway. The activation of these two proteases activates the downstream caspase‐3, which triggers apoptosis. After activation, both of them can activate downstream caspase‐3, which triggers apoptosis. Oxidative stress is one of the important reasons for the occurrence of apoptosis, and a large amount of ROS will cause metabolic disorders and organ failure and directly cause oxidative damage to lung parenchymal cells.^24^ FA was able to reduce the apoptosis level and ROS level of primary cells in the lung tissue of PF mice, reduce the MDA level, and increase the T‐SOD and GSH‐Px levels. Oxidative stress is usually inextricably linked to the fibrotic process in PF,^25^ and it has been found that the activation of oxidative stress‐related pathways in vivo advances the onset of tissue fibrosis and promotes the proliferation of fibroblasts.^26^ In the present study, we found that FA inhibited the expression level of the inflammatory factor IL‐6, and the expression of apoptosis‐related genes FasL, Fas, Bax/Bcl‐2, Cleaved caspase‐3, Cleaved caspase‐3/caspase‐3, suppressed the expression of oxidative stress‐related gene NOX4 in the lung tissues of mice with BLM‐induced PF, elevated the expression of antioxidant‐related genes HO‐1, CAT‐1, and p‐Nrf2/Nrf2 expression, and improved the pathological changes in lung tissues, suggesting that the improvement of PF by FA is related to the regulation of apoptosis and oxidative stress.

EMT is one of the main mechanisms of PF,^27^, ^28^ and the EMT process is regulated by several signaling pathways, including HIF‐1α signaling pathway, which is a key regulatory pathway for EMT initiation. During fibrosis, HIF‐1α upregulates the expression of VEGF and TGF‐β1,^29^ which in turn mediates the development of EMT.^30^, ^31^ Alveolar epithelial cells secrete large amounts of extracellular matrix (ECM) and vimentin during EMT,^32^ and at the same time promote the expression of α‐SMA, Collengen I, and inhibit the expression of E‐cadherin, which promote the extent of fibrosis.^33^, ^34^ In addition, A549 cells are derived from type II alveolar epithelial cells, and the alveolar epithelial cells undergo EMT under certain circumstances.^35^, ^36^ This study found that FA could regulate the abnormalities in proliferation levels, apoptosis levels, and ROS levels of A549 cells in an in vitro study, which in turn inhibited the development of EMT. In this study, we found that FA was able to decrease the expression levels of HIF‐1α, VEGF, TGF‐β1, Vimentin, α‐SMA, and collagen Ι, and increase the expression level of E‐cadherin, which suggests that the improvement of PF by FA is related to the EMT pathway.

In summary, FA can improve BLM‐induced PF in mice by regulating oxidative stress and cell apoptosis. This study suggests that FA may become a potential drug for treating PF, and the natural compounds discovered in this study are of great significance for drug development.

## 5 CONCLUSION

Our research suggests that the intervention effect of FA on BLM‐induced PF may be related to its modulation of oxidative stress and cell apoptosis levels, as well as the regulation of the EMT pathway in PF. These positive effects of FA may indicate its potential clinical significance in the treatment of PF.

## AUTHOR CONTRIBUTIONS

**Fan Yang**: Conceptualization; methodology; resources; software; visualization; writing‐original draft. **Qinqin Zhang**: Conceptualization; methodology; formal analysis; investigation; software. **Xi Wang**: Data curation; formal analysis; investigation; validation. **Yingbo Hu**: Data curation; investigation; visualization. **Suiqing Chen**: Conceptualization; methodology; software; funding acquisition; project administration; supervision.

## CONFLICT OF INTEREST STATEMENT

The authors declare no conflict of interest.

## ETHICS STATEMENT

All animal experiments have been approved by the Animal Ethics Committee of the Henan University of Chinese Medicine, Zhengzhou, China. Approval No. IACUC‐202307031.

## Data Availability

The original contributions presented in the study are included in the manuscript, further inquiries can be directed to the corresponding authors.
